# Complications of circumcision in male neonates, infants and children: a systematic review

**DOI:** 10.1186/1471-2490-10-2

**Published:** 2010-02-16

**Authors:** Helen A Weiss, Natasha Larke, Daniel Halperin, Inon Schenker

**Affiliations:** 1 MRC Tropical Epidemiology Group, Department of Epidemiology and Population Health, London School of Hygiene & Tropical Medicine, Keppel Street, London WC1E 7HT, UK; 2 Dept of Global Health and Population, Harvard School of Public Health, 665 Huntington St, Boston, MA, USA; 3 The Jerusalem AIDS Project, 4 Eliezer Hagadol Street, Jerusalem 91072, Israel

## Abstract

**Background:**

Approximately one in three men are circumcised globally, but there are relatively few data on the safety of the procedure. The aim of this paper is to summarize the literature on frequency of adverse events following pediatric circumcision, with a focus on developing countries.

**Methods:**

PubMed and other databasess were searched with keywords and MeSH terms including infant/newborn/pediatric/child, circumcision, complications and adverse events. Searches included all available years and were conducted on November 6^th ^2007 and updated on February 14th 2009. Additional searches of the Arabic literature included searches of relevant databases and University libraries for research theses on male circumcision.

Studies were included if they contained data to estimate frequency of adverse events following neonatal, infant and child circumcision. There was no language restriction. A total of 1349 published papers were identified, of which 52 studies from 21 countries met the inclusion criteria. The Arabic literature searches identified 46 potentially relevant papers, of which six were included.

**Results:**

Sixteen prospective studies evaluated complications following neonatal and infant circumcision. Most studies reported no severe adverse events (SAE), but two studies reported SAE frequency of 2%. The median frequency of any complication was 1.5% (range 0-16%). Child circumcision by medical providers tended to be associated with more complications (median frequency 6%; range 2-14%) than for neonates and infants. Traditional circumcision as a rite of passage is associated with substantially greater risks, more severe complications than medical circumcision or traditional circumcision among neonates.

**Conclusions:**

Studies report few severe complications following circumcision. However, mild or moderate complications are seen, especially when circumcision is undertaken at older ages, by inexperienced providers or in non-sterile conditions. Pediatric circumcision will continue to be practiced for cultural, medical and as a long-term HIV/STI prevention strategy. Risk-reduction strategies including improved training of providers, and provision of appropriate sterile equipment, are urgently needed.

## Background

An estimated one in three males worldwide are circumcised, with almost universal coverage in some settings and very low prevalence in others [1]. As with any surgical procedure, circumcision can result in complications [2-4]. The most common early (intra-operative) complications tend to be minor and treatable: pain, bleeding, swelling or inadequate skin removal. However, serious complications can occur during the procedure, including death from excess bleeding and amputation of the glans penis if the glans is not shielded during the procedure [5-10]. Late (post-operative) complications include pain, wound infection, the formation of a skin-bridge between the penile shaft and the glans, infection, urinary retention, meatal ulcer, meatal stenosis, fistulas, loss of penile sensitivity, sexual dysfunction and edema of the glans penis [11]. Circumcision is commonly conducted in neonates, infants and children for religious, cultural and medical reasons, yet there have been no systematic reviews of the published literature on complications associated with the procedure at this age.

Male circumcision is of public health interest as recent randomized controlled trials (RCT) have shown that adult circumcision reduces the risk of acquiring HIV infection by about 60% [12-14]. Several countries with high prevalence of HIV are now planning to expand access to safe circumcision [15], and the World Health Organisation (WHO) and the Joint United Nations Programme on HIV/AIDS (UNAIDS) have recommended considering neonatal circumcision in addition to adult circumcision as a longer-term HIV prevention strategy [16]. Pilot projects for neonatal and infant circumcisions are now being considered in several African countries, and to inform these programs, we undertook a systematic review of practices of paediatric circumcision, including prevalence, age at circumcision, types and training of providers, circumcision methods used, frequency of complications and cost. Since expansion of male circumcision for HIV prevention is recommended in regions with high rates of heterosexual transmission (in practice, much of southern and parts of eastern Africa), we carried out searches specifically for non-Western regions of the world. In this paper, we report findings of frequencies of adverse events associated with neonatal, infant and child circumcision.

## Methods

### Search strategy

PubMed, African Healthline, LILACS and the Cochrane Central Register of Controlled Trials databases were searched with keywords and MeSH terms including infant/newborn/pediatric/child, circumcision, complications adverse events, Africa, Asia and Arabic. For example, we searched PubMed with the following search terms: "Circumcision, Male" [Mesh] AND "Infant, Newborn" [Mesh] AND ("Africa" [Mesh] OR "Asia" [Mesh]); "complications " [Subheading] OR "Intraoperative Complications" [Mesh] OR "Postoperative Complications" [Mesh]) AND "Circumcision, Male" [Mesh] AND ("Africa" [Mesh] OR "Asia" [Mesh]); ("Child" [Mesh] AND "Circumcision, Male" [Mesh]) AND ("Africa" [Mesh] OR "Asia" [Mesh]); ("Infant, Newborn" [Mesh] OR "Child" [Mesh]) AND ("Circumcision, Male" [Mesh] OR ("Circumcision, Male/adverse effects" [Mesh] OR "Circumcision, Male/complications" [Mesh] OR "Circumcision, Male/contraindications" [Mesh] OR "Circumcision, Male/mortality" [Mesh])); "Circumcision" [Mesh] "Circumcision, Male " [Mesh] AND "Arabic".

Searches were conducted on November 6^th ^2007 and updated on February 14th 2009. There was no language restriction. We also searched reference lists of relevant papers, including a systematic review of complications of male circumcision in Anglophone Africa [17]. A total of 1349 published papers were identified through these searches. The abstracts of these papers were read and full copies of 223 papers with information on complications were obtained. Data were extracted by HW and NL into standardised forms in Access.

Infant and child circumcision is almost universal in the Arab world, and we conducted additional searches of the Arabic literature, including searches of relevant databases, book reviews in 10 key academic centres on Middle Eastern Studies and searches of the Hebrew University of Jerusalem libraries for Masters and PhD research thesis focused on male circumcision. Searches were conducted from June to August 2008. The Arabic literature searches identified 46 potentially relevant papers, of which six contained information on circumcision complications.

### Analysis methods and definitions

Hospital-based studies of circumcision-related complications are usually retrospective and record-based [9,18,19]. Complications in these studies are commonly recorded from discharge sheets, so tend to under-estimate the true frequency of complications because events occurring after discharge are not captured. Furthermore, not all post-operative complications will be seen again at the same hospital. We therefore present results separately for prospective and retrospective studies. Age at circumcision, and type of provider (medical or non-medical) were also thought *a-priori *to be associated with frequency of complications, and we present results stratified by these factors. We define neonatal as age up to 28 days, infant as 28 days-11 months, and child as 12 months-12 years. Many studies included boys circumcised at a range of ages. We included studies in which the mean or median age at circumcision was age 12 years or younger.

Definitions of complications varied between studies. To report complications as consistently as possible between studies, we excluded all cases of oozing or bleeding which was easily stopped by compression, as these were not consistently reported in all studies. Cases of excess residual foreskin or inadequate circumcision are also excluded - these are adverse outcome of circumcision and may involve further surgery, but are not medical complications *per se*. We also excluded some other minor complications from studies as noted under individual studies. We have also reported serious adverse events separately - these include complications defined as 'severe' or 'serious' by authors, or with long-term or life-threatening sequalae.

## Results

From the 223 potentially relevant papers, we identified 52 studies from 21 countries which included sufficient information to estimate frequency of adverse events following neonatal, infant and child circumcision. The remaining papers were largely case-reports and case-series of circumcision-related complications. We excluded one study among people with haemophilia [20], as any surgical procedure in haeomphiliacs is associated with a high risk of post-operative bleeding and is not representative of general populations.

### Complications following neonatal or infant circumcision

We identified 16 prospective studies of complications following neonatal and infant circumcision, from 12 countries [9-11,21-33] (Table 1). Of these, most used the Plastibell [11,22,23,25-28], with others using the Gomco clamp [21,24,30,32], freehand circumcision [9,31], or a combination of methods [27,29,33].

**Table 1:** Prospective studies of frequency of complications in studies of neonatal and infant circumcision

Author	Country	No. of patients	Age	Type of provider	Method	Follow-up period	**Frequency of adverse events**^**a**^	**Frequency of serious adverse events**^**b**^
Al Samarrai [11]	Saudi Arabia	2000	2-3 days	Junior staff with supervision	Plastibell	6 weeks plus immunisation clinic visits	1.4%^c^	0%

Amir^d ^[21]	Saudi Arabia	1000	Mean 9 days	Surgeon	Gomco clamp	1 year	1.6%	0%

Banieghbal[32]	South Africa	583	Neonatal	Surgeon	Gomco clamp	1 month	0.3%	0%

Ben Chaim [9]	Israel	19,478	Mean 8 days	83% Mohel 17% Physician	Freehand	-	0.1%	0.1%

Bhat [22]	Oman	250	Neonatal (min 1 day)	Paediatrician	Plastibell	-	0%	0%

Duncan [23]	Jamaica	205	Neonatal	Surgeon	Plastibell	1 week	1.5%	0%

Horowitz [24]	USA	130	98 neonatal 32 infants (3-8.5 months)	Pediatric urologist	Gomco clamp	3 days	Overall: 7.4% Neonatal: 0% Infants: 30%	0%

Manji [25]	Tanzania	368	7 days to 9 months	Pediatrician	Plastibell	-	2.8%^e^	0%

Mousavi [33]	Iran	586	<12 months	Surgeon	50% sleeve 50% Plastibell	-	Sleeve: 1.95% Plastibell: 3.1%^f^	Sleeve: 0% Plastibell 2.1%

Okafor [26]	Nigeria	102	Immediate post-partum	Experienced surgeon	Plastibell	1 year	0%	0%

Okeke^g ^[10]	Nigeria	322	8 days-13 months	55% Nurses 35% Doctors 9% Trad.	-	3 month	9.3%	1.0%

Osuigwe [27]	Nigeria	141	7-9 days	54% Doctors 44% Midwives 2% Trad. birth attendants	68% Plastibell 31% freehand	6 weeks	13.5% Plastibell: 8% Freehand: 27.3%	2.1%

Palit [28]	UK	1129	Mean age 11 weeks	Trained nurses under supervision of consultant urologists	Plastibell	3 months	5.5%	0.1%

Patel^d ^[29]	Canada	100	3-5 days old	98% Medical doctors 2% Traditional Providers	51% Gomco 47% Plastibell 2% Ritual	-	15%^h^	2%

Perlmutter [30]	USA	51	Neonatal	Obstetrician or resident	Gomco	Up to 2 hours	0%	0%

Rehman [31]	Pakistan	200	Infant	Surgeon	50% freehand 50% bonecutter	1 week	16%	0.5%

^a ^Cases of minor bleeding stopped with simple pressure or 'conservative management' and excessive foreskin/inadequate circumcision are not included

^b ^Includes complications defined as 'serious' or 'severe' by authors, or with long-term or life-threatening sequalae (partial amputation of glans, urethral laceration, need for re-surgery or plastic surgery)

^c ^18 patients with yellowish patches of sloughed tissue and erythema who did not have an infection confirmed through cultures, 4 patients with irregular skin margin and 4 patients with inadequate skin excision were excluded

^d ^In these studies patients who had undergone circumcision were identified retrospectively, but wherever possible patients were actively followed up to obtain accurate complication risks.

^e ^Risks by age at circumcision: 7-14 days: 0.9%; 15 days - 2 months: 4.7%; 2 - 9 months: 11.5%

^f ^Excludes 'excess mucosa' and 'delayed Plastibell falling off'

^g ^Patients were identified through an immunization clinic and a physical examination was conducted to confirm circumcision status and the presence and type of complications. Uncircumcised boys were followed up to identify boys circumcised at a later age and any complications

^h ^31 cases of mild oozing, 7 cases of mild infection with no antibiotic treatment were excluded

The median frequency of any adverse event was 1.5% (range 0-16%), and median frequency of any serious adverse event was 0% (range 0-2%). Nine studies reported no serious adverse events, but three studies reported that 1-2% of boys had a serious complication [10,27,29]. One, a Canadian study of 100 neonates circumcised in 1961/1962 using the Gomco clamp or Plastibell reported one severe infection requiring antibiotics and one severe meatal ulcer [29]. Less severe complications were reported in a further 13 boys in this study. The other two studies with serious complications were from Nigeria. In one, among 141 boys circumcised in 3 hospitals in southeast Nigeria, complications were assessed at a 6 week post-operative visit or if they presented earlier with any complication [27]. Three boys (2.1%) had a urethral laceration. The most common complications in this study were minor including bleeding (9%) and meatal stenosis (3.5%). Complications were substantially more common when circumcision had been performed freehand (27% excluding incomplete circumcision) rather than using the Plastibell (8%), and when performed by midwives (19%) rather than doctors (7%). Moreover, among the doctors, the reported frequency of complications at the public (University Teaching) hospital was 1.6%, compared to 20% at private hospitals where the level of training and supervision is lower. A much higher frequency (90%) was seen at the mission hospital, which acts as a referral centre for complicated circumcisions. Three circumcisions had been performed by a traditional birth attendant, and all three had resultant complications (one bleeding, one incomplete circumcision, and one urethral fistula). The other study was among 322 infants attending a welfare clinic in Ibadan [10], in which there were 2 cases of amputation of the glans penis and one buried (trapped) penis. Overall in this study, complications were reported in 9.3% of boys, with a further 11% having excess residual foreskin. The most common complication was excessive loss of foreskin (n = 16; 5%). Unusually, no cases of bleeding, wound infection, or haematoma were reported in this study. The method used was not reported for the majority of infants, and complications were most frequent when the procedure was performed by nurses rather than doctors or traditional circumcisers (data not given).

Of the remaining 13 studies, five reported adverse events in 0.3% or fewer boys [9,22,26,30,32], four in around 2% [11,21,23,25], and the remaining four studies reported adverse events in up to 16% of boys [24,28,31,33]. The studies with highest frequency of complications are from Pakistan and the United Kingdom (UK). The study from Pakistan reports on 200 infants circumcised under local anaesthesia at a Military Hospital using either the freehand or bone-cutter method (a forceps-guided method which does not shield the glans) [31]. Bleeding (defined as requiring more than an application of a pressure bandage) was reported in 9% of boys, and 7% had a local infection of the skin and mucosa. In the UK study, 1129 infants were circumcised by nurses using the Plastibell under local anaesthesia [28], and overall 125 (11.1%) of infants required some degree of follow-up, with complications seen in 5.5%. The most common complication involved the Plastibell ring device itself (3.6%), which is left on after the procedure and normally takes 7-10 days to fall off. The problems included delayed separation of the ring, incomplete separation of the ring, or the ring becoming stuck on the penile shaft. In all cases, the ring was removed without need of anaesthesia and the authors report this removal was quick, simple and atraumatic.

Three studies reported substantial variation in complication frequencies by age or circumcision method. For example, a US study of circumcision by the Gomco clamp stratified by age at circumcision and found no complications in 98 boys circumcised neonatally, but that 12/32 (30%) of infants aged 3-8.5 months had postoperative bleeding requiring suture repair [24]. These 32 boys were circumcised under general anaesthesia and no complications from the general anesthesia were reported. In another study, complications were seen more frequently using the Plastibell (12/381; 3.1%) than the sleeve technique (4/205; 1.95% [33]).

A further ten studies on neonatal/infant circumcision were retrospective hospital-record based studies (Table 2). Five of these were from the USA, two from Pakistan, one each from Israel, Oman and Turkey. Reported frequency of complications were slightly lower than for the prospective studies, with five studies finding very low frequencies (≤0.6%) [19,34-37] and four in the range 2-4% [38-41]. The study reporting the highest proportion (4% in neonates, 10% in infants) included late complications (most commonly foreskin adhesions (7.8%)), with 3 cases (1.3%) of meatitis and 3 requiring circumcision revision (1.3%) [42]. As with prospective studies in neonates and infants, few serious adverse events were reported (<0.2% in all studies except among infants in one US study, where 3/230 (1.3%) of infants required circumcision revisions [42]).

**Table 2:** Retrospective studies of frequency of complications in studies of neonatal and infant circumcision

Author	Country	Year of study	Number of patients	Age	Type of provider	Method used	**Frequency of adverse events**^**a**^	**Frequency of serious adverse events**^**b**^
Al-Marhoon [34]	Oman	1997-2000	171	Neonatal	Surgeon	Plastibell	1.2%^c^	0% (Two needed sutures)

Christakis [35]	USA	1987-1996	130475	Neonatal	-	-	0.2%	0.2%

Eroglu [41]	Turkey	2001-2002	214	Neonatal	Surgeon	Gomco clamp	2.3%	0% (One needed sutures)

Gee [38]	USA	1963-1972	5521	Neonatal	Supervised medical student, resident, or physician	52% Gomco clamp 48% Plastibell	1.7% Gomco 2.3% Plastibell	0.2%^d^

Iftikhar [36]	Pakistan	1998-2001	316	0-12 yrs (72% within 1 week of birth)	Pediatric surgeon	Plastibell (<2 yrs old) Open technique (≥ 2 yrs)	0.6%	0%

Metcalf [42]	USA	1974-1979	591	61% Neonatal 39% Post-neonatal	-	-	4% neonatal 10%% infants^e^	0.3% neonatal 1.3% infants

O'Brien [39]	USA	1985-1986	1951	Neonatal	-	43% Gomco 9.5% Plastibell 14.5% Electrocautary 33% not specified	3.1% overall	0%

Rafiq [40]	Pakistan	2000	100	Neonatal	Surgeon	Plastibell	2%	0%

Shulman [37]	Israel	1955-1963	8000	Neonatal	Mohelim	-	0.3%	0.1%

Wiswell [19]	USA	1980-1985	100157	Neonatal	Surgeons		0.2% 'serious'	0.2%

^a ^Cases of minor bleeding stopped with simple pressure or 'conservative management' and excessive foreskin/inadequate circumcision are not included

^b ^Includes complications defined as 'serious' or 'severe' by authors, or with long-term or life-threatening sequalae (partial amputation of glans, urethral laceration, need for re-surgery or plastic surgery)

^c ^Excludes one patient unable to pass urine for 24 hours

^d ^The authors note that 14 patients (0.2%) had 'really significant' complications - one life-threatening haemorrhage, 4 systemic infections, 8 circumcisions of infants with hypospaidas, and one complete denudation of the penile shaft.

^e ^6 patients with hygiene concerns were excluded

### Complications following child circumcision by medically trained providers

We identified ten prospective studies of complications in children aged one year old or older following circumcision by medically trained providers (Table 3) [43-52]. The median frequency of any adverse event was 6% (range 2-14%), and median frequency of any serious adverse event was 0% (range 0-3%). Adverse events were seen most commonly among boys circumcised mainly for medical, rather than religious or cultural, reasons. In one of these studies, among boys circumcised in the UK mainly for phimosis, 4/140 boys (2.8%) required acute re-admission to hospital [49] and the frequency of any adverse events was 6.4%. In the other, a Danish study of boys circumcised mainly for balanitis or phimosis, 1/43 (2.3%) boys required re-operation following Plastibell circumcision [52]. Of the other studies, in which boys were circumcised mainly for non-medical reasons, two studies reported any adverse event in about 2% of boys [43,50], three were 2-5% [47-49], and higher frequencies (7-14%) were seen in studies from the Netherlands [51], India [44], Iran [45] and Turkey [46]. Complications included bleeding, infection, meatal stenosis and problems with the Plastibell device. The study from the Netherlands reported on complications among 94 Muslim boys circumcised under local anaesthesia outside the hospital. Of these, 13 were seen again because of bleeding (n = 4), infection (n = 2) or swelling (n = 7) [51], excluding the two cases of mild bleeding the frequency of complications was 12%. The Indian study was also small (n = 15) and reported 2 cases of minor wound separation which did not require further surgical intervention [44]. The study from Iran was an RCT in 394 boys, in which 13 (3.3%) boys developed meatal stenosis and 26 (6.6%) had infections at the circumcision site, and 43 (10.9%) had post-circumcision bleeding. Complications were significantly less frequent among boys who parents were randomised to use a lubricant (petroleum jelly) on the circumcision site [45]. Finally, the Turkish study reports complications following a hospital-based mass circumcision exercise, in which 700 boys were circumcised over 5 days. Excluding the cases of bleeding stopped by simple compression, 8% of boys had a complication, most commonly infection (2.7%) and inadequate foreskin removal accompanied by secondary phimosis (2.1%).

**Table 3:** Prospective studies of frequency of complications in studies of child circumcision undertaken by medical providers

Author	Country	Years	Setting	N	Age	Provider	Method	Indication	Follow-up period	**Frequency of adverse events**^**a**^	**Frequency of serious adverse events**^**b**^
Ahmed [43,59]	Comoros Islands	1997-1998	Home	3824	2-8 years	Surgical aids, nurses & midwifes	Dorsal slit	Routine	11 days	2.3%	0.5%

Aldemir [48]	Turkey	2006	Hospital	200	2-9 years	Urologist	65% Smart clamp 35% Dissection	Routine	6 weeks	5%	1%

Bazmamoun [45]	Iran	2006-2007	Hospital	394	Mean 9 months	Surgeon	Sleeve	Routine	6 months	7-10%^c^	0%

Griffiths [49]	England	1985	Hospital	99	Mean 4.3 years	-	Dissection	85% medical 11% religious 4% other	3-5 weeks	6.4%^d^	2.8%^e^

Ozdemir [46]	Turkey	1990s	Mass circ. in hospital	700	8 days to puberty	-	Forceps guided	Routine	3 months	8%^f^	0%

Schmitz [51]	Holland	1997	Health centre	94	Median 3 years	GP residents under supervision of a surgeon	Freehand	Religious	1 week	12%	0%

Schmitz [50]	Malaysia	2001	Community	64	Median 10 years	Medical assistants supervised by doctors	TaraKlamp	Routine	6 weeks	1.6%	0%

Sharma [44]	India	2003	Hospital	15	2-25 years	Surgeons	Dorsal slit	Medical or religious	90 days	13.3%	0%

Sorensen [52]	Denmark	1981-1983	Hospital	43	Mean 6.5 years (range 1-13)	Surgeon (early stage in training)	Plastibell	Medical	Mean 29 months	Immediate postoperative (reported) 9.3%^g^ Late complications (reported) 0%^h^	0%

Subramaniam [47]	Singapore	-	Hospital	152	Mean 7 years	Surgeon	CO_2 _laser	Not given	-	4.6%	0.7%

^a ^Cases of minor bleeding stopped with simple pressure or 'conservative management' and excessive foreskin/inadequate circumcision are not included

^b ^Includes complications defined as 'serious' or 'severe' by authors, or with long-term or life-threatening sequalae (partial amputation of glans, urethral laceration, need for re-surgery or plastic surgery)

^c ^13 boys had meatal stenosis and 26 had infection. It is not clear whether there is overlap between these two groups.

^d ^Defined by the authors as any admission to hospital or further surgery.

^e ^Acute re-admissions to hospital

^f ^Includes 15 cases of inadequate circumcision, since these were accompanied by secondary phimosis

^g ^One case of haemorrhage that stopped spontanesouly, 2 cases of erythema and pus with no confirmed infection or antibiotic treatment and 24 cases of dysuria due to irritation of the meatus due to the presences of a Plastibell excluded

^h ^Seven cases of slight irritation of the glans excluded

Adverse events in 11 retrospective studies tended to be less frequent than for the prospective studies, probably due to under-ascertainment of complications. Most studies reported no serious adverse events (Table 4), but one [53] reported that 2/79 (2.5%) boys required circumcision revisions following circumcision by the Plastibell device. Frequencies of any adverse event varied from 0.3% in a study from Nigeria (5 minor complications reported out of 1563 circumcisions in the hospital over a 15 year period [7]) to 12% (15/129) in South Africa (mostly bleeding, haematoma and infection) and 17.5% (28/160) among boys circumcised with a new disposable device (the Shenghuan Disposable Minimally Invasive Circumcision Anastomosis Device) in China (mainly mild oedema (10%) but also moderate oedema and 2 cases of infection).

**Table 4:** Retrospective studies of frequency of complications in studies of child circumcision undertaken by medical providers

Author	Country	Years	Setting	N	Age	Method used	Indication	**Frequency of adverse events**^**a**^	**Frequency of serious adverse events**^**b**^
Ahmed [7]	Nigeria	1981-1995	Hospital	1563	Mean 4 years	-	Routine	0.3%	-

Atikeler [54]	Turkey	1999-2002	Hospital	782	Mean 6 years	-	Medical indication or religious reasons	2.6%	0%

Cathcart [74]	UK	1997-2004	Hospital	66519	0-15 years	-	98% Medical	1.2%	0%

Lazarus [53]	South Africa	1999-2005	Hospital	95	'boys'	-	Medical or religious	5.1%	2.5%

Leitch [69]	Australia	1960s	Hospital	200	Mean 2 years	-	71% Medical 29% Cultural	11%	0%

Millar [75]	South Africa	1985-1987	Hospital	129	3 months to 10 years	Plastibell	19 revisions	12%	-

Ozdemir [46]	Turkey	1990s	Hospital	600	8 days to puberty	Forceps guided?	Routine	1.7%	0%

Peng [76]	China	2005-2007	Hospital	160	5-12 years	Shenghu disposable device	Mainly medical	Complications whilst wearing device : 17.5%^c^ Complications after removal of device : 0.6%	0.6%

Rizvi [64]	Pakistan	1981-1991	Hospital	3096	'children'	-	-	1.6%	-

Wiswell [18]	USA	1985-1992	Hospital	476	Mean 3 years	Freehand or sleeve	Cultural (67%) Medical (33%)	1.7%	0.2%

Yegane [77]	Iran	2002	Community	1766	71% after 2 years of age	-	-	4.6% overall (late complications) 2.8% Urologists/surgeons 6.1% GPs/pediatricians 9.1% Paramedics	0%

^a ^Cases of minor bleeding stopped with simple pressure or 'conservative management' and excessive foreskin/inadequate circumcision are not included

^b ^Includes complications defined as 'serious' or 'severe' by authors, or with long-term or life-threatening sequalae (partial amputation of glans, urethral laceration, need for re-surgery or plastic surgery)

^c ^Seventy cases of swelling pain from nocturnal erection excluded

### Complications following child circumcision by non-medically trained personnel

Table 5 summarizes the five studies of complications following circumcision by non-medically trained providers. In these studies, frequencies of adverse events are generally higher, and complications more serious, even including penile amputation [7]. A high frequency of complications was seen in a retrospective study from Turkey of 407 boys circumcised at two traditional mass circumcision events [54]. The mean age of the boys at time of circumcision was 7 years (range 1-14 years) and the procedure had taken place in non-sterile conditions by unlicensed providers. Overall, complications were seen in 73% of boys, with the most common complications being wound infection (14%), subcutaneous cysts (14%), bleeding which needed suturing (12%), and haematoma (6%). Five boys (1.3%) developed a urinary infection requiring hospitalisation and intravenous antibiotics. A further 12% of boys were deemed to have incomplete circumcision. In addition, 3 patients with (contra-indicated) hypospadias had been circumcised indicating inadequate screening of the boys.

**Table 5:** Retrospective studies of frequency of complications in studies of child circumcision undertaken by non-medical providers

Author	Country	Years	Setting	Number of males	Age at circumcision	Provider	**Frequency of adverse events**^**a**^	**Frequency of serious adverse events**^**b**^
Ahmed [7]	Nigeria	1981-1995	Community	1360 (approx)	Mean 4 years	Traditional	3.4%	-

Atikeler [54]	Turkey	1999-2002	Community	407	Mean 7 years	Traditional	73%^c^	

Lee [55]	Phillipines	2002	Community	114	42% 5-9 years 52% 10-14 years 5% 15-18 years	32% medical 68% traditional	63%^d^	3.5%

Myers [56]	Nigeria	-	Community	750	Infant/child	68% traditional 25% nurse/midwife 4% doctor	2.8%	-

Yegane [77]	Iran	2002	Community	1359	71% after 2 years of age	Traditional circumcisers	2.7%% (late complications)	0%

^a ^Cases of minor bleeding stopped with simple pressure or 'conservative management' and excessive foreskin/inadequate circumcision are not included

^b ^Includes complications defined as 'serious' or 'severe' by authors, or with long-term or life-threatening sequalae (partial amputation of glans, urethral laceration, need for re-surgery or plastic surgery)

^c ^This very high rate of complications consisted of bleeding (24%), infection (14%), incomplete circumcision (12%), subcutaneous cysts (15%), haematoma (6%), ischaemia (3%), penile adhesion (3%), and other conditions. Of the 97 cases of bleeding, 48 could not be stopped by haemostatic bandage and were sutured. Infections were treated with parenteral or oral antibiotics.

^d ^Of these,94% were reported swollen or inflamed penises. Four respondents (3.5%) of those circumcised) reported profuse bleeding

The retrospective study from the Philippines interviewed 114 males aged 13-51 (mean age 25.9 years), of whom 94% reported having been circumcised below the age of 14 years. Most (68%) had been circumcised by non-medical personnel, and 60% of participants reported post-circumcision complications (inflammation and swelling) to their circumciser, and 4 (3.5%) reported profuse bleeding [55]. In contrast, in a household-based study in southwest Nigeria, respondents reported very few complications (2.8%) following circumcision, mainly by traditional providers [56]. Among 750 child circumcisions, there were 12 cases reported of excessive bleeding, 6 infections, 2 cases of tetanus and one death. The authors report that, although they include the death, there was insufficient information to be certain it was caused by circumcision. A study from Iran reported a late-phase complication frequency of 2.7% following traditional circumcision and a further 5% had excessive residual foreskin. This was similar to circumcisions performed by urologists or surgeons (2.8%), but lower than for GPs/paediatricians (6.1%) or paramedical personnel (9.1%). The authors argue that this is because traditional circumcisers in Iran are experienced and paramedical personnel do not receive effective training.

## Discussion

Male circumcision is a common surgical procedure, but few epidemiological studies have reported frequency of adverse events, most commonly bleeding and infection. Our review shows that serious adverse events are rare, but there is wide variation in reported frequencies of adverse events following circumcision. This is likely to be due to several factors directly associated with complications such as age at circumcision, training and expertise of the provider, the sterility of the conditions under which the procedure is undertaken and the indication (medical/cultural) for circumcision. In addition, there is variation due to methodological issues such as duration of follow-up, epidemiological study design, and definition of complications.

In general, complications (reported by parents) occur least frequently among neonates and infants than among older boys, with the majority of prospective studies in neonates and infants finding no serious complications, and relatively few other adverse events, which were minor and treatable. The prospective studies in older boys also found virtually no serious adverse events, but a higher frequency of complications (up to 14%) even when conducted by trained providers in sterile settings [47]. The lower frequency of complications among neonates and infants is likely to be attributable to the simpler nature of the procedure in this age group, and the healing capability in the newborn. Further, a major advantage of neonatal circumcision is that suturing is not usually necessary, whereas it is commonly needed for circumcisions in the post-neonatal period. This advantage is illustrated by the US study in which no complications were seen among 98 boys circumcised in the first month of life, but 30% of boys aged 3-8.5 months had significant postoperative bleeding [24]. There are alternatives to suturing, either with the disposable clamps, or with alternatives such as cynoacrylate glue [44] and further research in this area is needed.

Several studies stress the importance of careful training and experience of the provider, and the sterility of the setting. This was most clearly noted in a Nigerian study [27] in which 24% of boys had reported complications (including retention of excess residual foreskin), but only 1.6% of those circumcised at the public (University Teaching) hospital by medical doctors. Similarly, two case-control studies from Israel have found that UTI are 3-4 times more likely to occur following circumcised by a traditional, rather than medical provider [57,58]. However, as noted in our review, neonatal circumcision following traditional circumcision in Israel has low complication rates overall [9]. A further example is the study from the Comoros Islands which reported results of an exercise in which specific training had been given to surgical aids and nurses to perform circumcisions. The proportion of boys with complications (2.3%) was reported to be a great improvement on that by traditional non-medically trained providers [43,59]. The high frequency of adverse events following circumcision by untrained providers in non-sterile settings is striking in two studies of traditional circumcision which found alarmingly high prevalence of around 80% [54,60]. Notably, in one of these, the self-reported frequency was much lower, illustrating the under-ascertainment that can occur in retrospective studies. Mass circumcisions are particularly risky, even when undertaken in the hospital. For example, the Turkish study of 700 children circumcised during a 5 day period recorded a complication frequency of 8%, likely due to the difficulty in providing sufficient sterile equipment and conditions [46]. The reason for surgery can also influence the risk of adverse events as seen in the studies of child circumcision where more complications were generally seen if circumcision was conducted for medical rather than religious reasons.

Our systematic review was restricted to circumcision complications among boys aged 12 years or under. However, there are several published studies of circumcision complications among adolescent and adult men (Table 6) and these indicate a generally higher frequency of complications than seen in neonates, infants and children. In the three RCTs of circumcision in adult men, complications were observed in 2-7% of HIV-negative men [14,61,62], and in 6-8% of HIV positive men [14,62]. The most detailed observational study was conducted among the Babukusu ethnic group in western Kenya. Of 562 adolescents circumcision by a medical provider (or reported as such), 18% had a complication, as did 35% of boys circumcised traditionally [60]. A sub-study in the same population directly observed 24 boys undergoing medical and traditional circumcision respectively and found that of those circumcised medically, only one boy had no adverse events, and 3 permanent adverse sequalae were reported, including one very serious life-threatening case by a 'medical' practitioner who was later found to have no documented medical qualifications [60]. Among the 12 directly observed traditional circumcisions, complications were seen in 10 boys (83%), and 4 (33%) were judged to have permanent adverse sequelae. None had fully healed by 30 days post-operation. Detailed examination showed that traditional circumcision was also associated with slower healing, more swelling, laceration and keloid scarring [60]. These results show that under non-sterile conditions, adolescent and adult circumcision can frequently be associated with severe complications. Other case-series of circumcision complications among adolescents and young men also report severe morbidity and mortality [63-68]. Reported complications tend to be more common in this age group than for neonates and infants, even when circumcision is conducted under the 'gold standard' conditions such as in the RCTs.

**Table 6:** Frequency of complications in studies of adolescent and adult circumcision

Author	Country	Years	Setting	N	Age	Provider	Method	Indication	Follow-up period	**Frequency of adverse events**^**a**^	**Frequency of serious adverse events**^**b**^
Auvert [14]	South Africa	2002-2004	GP offices	1495 HIV neg	18-24 years	GPs	Forceps guided	Enrolled in trial	1 month	3.6%	-

Auvert [14]	South Africa	2002-2004	GP offices	73 HIV positive	18-24 years	GPs	Forceps guided	Enrolled in trial	1 month	8.2%	-

Bailey [60]	Kenya	2004	Home or community	445	66% aged below 15 years	Traditional	-	Cultural	30-89 days	35%	24%^c^

Bailey [60]	Kenya	2004	Home or community	12		Traditional	-	Cultural	~3 months	83%	33%^d^

Bailey [60]	Kenya	2004	Hospital, health centre, or private office	562	90% aged below 15 years	Clinician^e^	-	Cultural	30-89 days	18%^f^	19%^h^

Bailey [60]	Kenya	2004	Hospital, health centre, or private office	12	-	Clinician^j^	-	Cultural	~3 months	92%^e^	25%^i^

Bowa [78]	Zambia	2004-2006	Urology outpatient clinic	900	5 months to 96 years	Trained clinical officer	Dorsal slit method	Cultural	8 weeks	3.0%	0.06% at 8 weeks

Kigozi [62]	Uganda	2003-2005	Trial operating theatre	2326 HIV neg	15-49 years	Trained physician	Sleeve method/	Enrolled in trial	6 weeks	7.4%	0.2% severe 3.3% moderate

Kigozi [62]	Uganda	2003-2006	Trial operating theatre	420 HIV positive	15-49 years	Trained physician	Sleeve method/	Enrolled in trial	6 weeks	6.0%	0% severe (3.1% moderate)

Krieger[61]	Kenya	2002-2005	Trial clinic	1475	18-24 years	Medical and clinical officers	Forceps guided	Enrolled in trial	90 days	1.8%	0% severe (0.7% moderate)

Magoha [79]	Nigeria & Kenya	1981-1998	Hospital	249	32% neonates 6% children 61% adolescent/adult	Surgeon	Forceps guided	72% Cultural/religious 12% Parental request 16% Medical	-	11%	2.8% severe^g^

Peltzer[80]	South Africa			78	Median 19 years (range 16-25)	Doctors and nurses following 1 day training		Cultural (Xhosa initiat	-	3.8%	0%

^a ^Cases of minor bleeding stopped with simple pressure or 'conservative management' and excessive foreskin/inadequate circumcision are not included

^b ^Includes complications defined as 'serious' or 'severe' by authors, or with long-term or life-threatening sequalae (partial amputation of glans, urethral laceration, need for re-surgery or plastic surgery)

^c ^Wound not healed at 60 days after surgery

^d ^Permanent adverse sequale

^e ^Anyone considered by the participant to be a clinician

^f ^Including an unknown number with residual foreskin

^g ^Includes severe haemorrhage (n = 3), scrotal laceration (n = 2), penile shaft denudation (n = 1) and glandular injury (n = 1).

A major challenge in our review was to standardise the definition of complications. For example, Okeke et al [10] report complications in 20% of boys, of which half were excessive residual foreskin - an adverse event but arguably not a medical risk. We excluded these cases where possible. Similarly, the paper by Gee et al [38] cites a total of 110 complications out of 5521 (2.0%) but states that only 14 complications (0.2%) were considered 'really significant' (one life-threatening hemorrhage, 4 systemic infections, 8 circumcisions of infants with hypospadias and one complete denudation of the penile shaft). The other complications included bleeding, infection, circumcision of hypospadiasis, and a Plastibell ring that was too tight. The problem of defining complications is also highlighted in the early (1961-1962) study from Canada in which moderate or severe complications (bleeding, infection, meatal ulcer, meatal stenosis and phimosis) were seen in 15 infants (15%) but a further 68 infants had mild bleeding, meatal ulcers or infection [29]. Complication risks in this study have previously been reported as 55% [4], which includes any bleeding, including oozing. A further example is the Australian study [69] which reported complications in 8% of boys, which included several cases of mild bleeding which either ceased spontaneously or with simple management such as digital pressure. We have attempted to report 'severe' or 'serious' adverse events as a separate outcome, but data on this is often limited and it would be useful to produce a standard classification of mild, moderate and severe complications following circumcision so that in future studies may be more easily comparable. Other limitations related to the design of the epidemiological studies. The length of follow-up varies between, and within, studies, and may affect the estimated frequency of complications. For this reason we tend not to term the frequency as a 'risk'. It is also possible that the lower frequencies of complications in prospective studies are due to improved procedures by practitioners or improved hygiene by patients as a result of participating in the study. Finally, a number of studies are small and the estimates of frequency of complications will be correspondingly imprecise.

We excluded one study of circumcision among patients with inherited bleeding disorders [20] as we were interested in complications in general populations. In this study, of 71 patients diagnosed from 1961-1996, 52% had a record of post circumcision bleeding. In many settings, boys are not asked about a family history of bleeding disorders and this can potentially lead to severe circumcision-related complications.

## Conclusion

Male circumcision is commonly practiced and will continue to occur for religious, cultural and medical reasons. In general, complications are minor and treatable, especially at young ages, but high frequency of complications, and severe complications, are seen when the procedure is undertaken by inexperienced providers, in non-sterile settings or with inadequate equipment and supplies. Further prospective studies with monitoring of risks following circumcision are needed to document complications using standardised definitions, and to compare the risks associated with different methods, age at circumcision, and to evaluate the impact of specific and ongoing training of providers. Such studies are underway in some settings where male circumcision services are being expanded for HIV prevention. A set of guidelines on expansion of male circumcision have been produced by WHO/UNAIDS, and include operational guidance for scaling up male circumcision for HIV prevention, a surgical manual for male circumcision under local anaesthesia, guidance for decision-makers on human rights, ethical and legal considerations protocols for monitoring and evaluation [70].

There is a clear need to improve safety of male circumcision at all ages through improved training or re-training for both traditional and medically trained providers, and to ensure that providers have adequate supplies of necessary equipment and instruments for safe circumcision. Strategies for training and quality assurance are needed and will be context specific. In Swaziland, "Operation AB" demonstrated a comprehensive model of training teams of medical providers in safe and swift adolescent and adult circumcisions, with improved sterilization equipment and clients' education, at community-level clinics [71] In Ghana, where neonatal circumcision is almost universal, the formal Health Service provides training to traditional providers in Accra, with training on basic hygiene and provision of necessary equipment, such as sterile gloves and dressings [72]. In South Africa it has been suggested that community health nurses create opportunities to educate traditional circumcisers of adolescents and adults on basic hygiene requirements to be met before, during and after circumcision [72], USAID/PATH/MSH have designed a training program in the Eastern Cape for training traditional providers about safe circumcision practices [73]. Links between the formal and informal health sectors should be explored elsewhere to institute quality standard practices for both traditional and medical circumcisers, for example wearing sterile gloves, using sterile instruments and appropriate aftercare, and creating a formal structure through which to monitor and regulate the conduct of circumcision. Through these steps, it is likely that the safety of this common procedure can be substantially improved.

## Abbreviations

GP: General Practitioner; RCT: Randomised controlled trials; UK: United Kingdom; UNAIDS: The Joint United Nations Programme on HIV/AIDS; USA: United States of America; WHO: World Health Organisation.

## Competing interests

The authors declare that they have no competing interests.

## Authors' contributions

The review was designed and conducted by HW and NL. The first draft of the paper was written by HW. IS and DH critically reviewed the manuscript and approved the final version. All authors read and approved the final version of the paper.

## Pre-publication history

The pre-publication history for this paper can be accessed here:

http://www.biomedcentral.com/1471-2490/10/2/prepub
